# Evaluation of link between COVID-19 adjacent spike in hydroxychloroquine use and increased reports of pemphigus: a disproportionality analysis of the FDA Adverse Event Reporting System

**DOI:** 10.3389/fimmu.2024.1470660

**Published:** 2024-12-20

**Authors:** Justin Baroukhian, Kristina Seiffert-Sinha, Kristopher Attwood, Animesh A. Sinha

**Affiliations:** ^1^ Department of Dermatology, Jacobs School of Medicine and Biomedical Sciences, Buffalo, NY, United States; ^2^ Department of Biostatistics and Bioinformatics, Roswell Park Cancer Institute, Buffalo, NY, United States

**Keywords:** hydroxychloroquine, COVID-19, pemphigus, autoimmunity, exposome, FAERS, environmental factors, autoimmune bullous disease

## Abstract

**Importance:**

Identifying environmental factors that contribute to disease onset/activity in PV stands to improve clinical outcomes and patient quality of life by strategies aimed at reducing specific disease promoting exposures and promoting personalized clinical management strategies.

**Objective:**

To evaluate the association between hydroxychloroquine use and the development of pemphigus using population level, publicly available, FDA-generated data.

**Design:**

Observational, retrospective, case-control, pharmacovigilance analysis.

**Setting:**

Population based.

**Participants:**

Individuals who either independently or via their healthcare provider submitted a voluntary report of a drug related adverse event to the FDA from Q4 of 2003 to Q2 of 2023.

**Exposure:**

Cases were identified by the presence of adverse events described by the MedDRA preferred term (PT) of “pemphigus” (10034280) and then sorted based on exposure to the drug of interest, hydroxychloroquine, or lack thereof.

**Main outcomes and measures:**

Frequency of hydroxychloroquine exposure among those individuals who reported an adverse event of pemphigus to the FDA; quantification of the reporting odds ratio (ROR).

**Results:**

We identified a total of 2,548 reports that included the adverse event pemphigus; among these, 1,545 (n=706 (41.92%) age 18-64, n=1 age 65-85 years, and n=977 (58.02%) with no age specified; n=1,366 (81.12%) females, n=4 (0.24%) males, and n=314 (18.65%) with no gender specified) included exposure to hydroxychloroquine (ROR, 282.647; 95% CI, 260.951-306.148). We then stratified those reports that included the combination of pemphigus and hydroxychloroquine by gender and found that while the association between the exposure and adverse event remained significant across genders, the magnitude of the effect sizes differed significantly (p<0.001), being over 100-fold greater among females (ROR, 378.7; 95% CI, 339.0-423.1) compared to males (ROR, 3.6; 95% CI, 1.4-9.8).

**Conclusions and relevance:**

The frequency of reports containing the combination of the adverse event pemphigus and exposure to the drug hydroxychloroquine was disproportionately elevated across all genders in the years since the start of the COVID-19 pandemic. The disproportionately elevated frequency of reports of the combination of pemphigus and hydroxychloroquine supports an association between the two, corroborates previous case-report based evidence for such an association, suggests that hydroxychloroquine represents a possible trigger factor for the development of pemphigus, and paves the way for future research that is capable of establishing causality.

## Highlights

- Question: Can an association between hydroxychloroquine use and the development of pemphigus, previously reported in a single case report, be corroborated using population level data?
- Findings: In this observational, retrospective, case-control, pharmacovigilance analysis of the FDA Adverse Event Reporting System, odds of reporting the adverse event pemphigus were significantly elevated, over 200-fold, among individuals exposed to hydroxychloroquine and nearly twice that in females.
- Meaning: Environmental exposures such as drugs are relevant players in the pathogenesis of autoimmune diseases; drug triggered autoimmunity is an entity with particular relevance to dermatology, and hydroxychloroquine likely represents a drug trigger of pemphigus.

## 1 Introduction

The World Health Organization (WHO) defines pharmacovigilance as the science and activities relating to the detection, assessment, understanding, and prevention of adverse events and drug related problems (1). The FDA Adverse Event Reporting System (FAERS) is a pharmacovigilance database developed by the Food and Drug Administration (FDA) to aid in post-marketing drug safety surveillance. The database consists of voluntary reports from either healthcare professionals or consumers. It is the largest such database in the world, with over 11 million reports at the time of authorship (2). Disproportionate reporting of a particular combination of drug exposure and adverse event, as revealed and quantified by disproportionality analysis, is instrumental in helping to decipher whether the reports seen for a particular drug-event combination are the results of mere chance or more likely to have been caused by the drug of interest (3). The development of autoimmunity as an adverse event following drug exposures is well known, but continues to be elucidated for the spectrum of specific drug-disease interactions.

Based on the multifactorial complexity associated with the etiology of autoimmune diseases, it is well established that both genetic and non-genetic elements, including potential drug triggers are implicated in the disruption of immune self-tolerance (4–6). Pemphigus vulgaris (PV), is a prototypic organ specific human autoimmune disease that presents clinically with fragile, superficial blisters that readily rupture, affecting skin surfaces and/or mucous membranes. The hallmark intraepidermal acantholysis that histologically characterizes PV is linked to the action of autoantibodies targeting critical desmosomal constituents—desmoglein 3 (Dsg3), and in many cases also desmoglein 1 (Dsg1)—, and potentially other non-Dsg targets (7–10) that are ultimately integral to cellular adhesion in the epidermis (11). The mainstay of treatment in PV remains in large part centered on general immunosuppression with systemic steroids, and non-steroidal immunosuppressives.

Robust evidence underscores a genetic component in predisposing to pathogenesis, with over 90% of individuals with PV of Caucasian descent carrying either the DRB1*0402 or DQB1*0503 class II HLA alleles (12, 13). Non-HLA genes linked to PV remain to be identified. While required, genetic factors are not sufficient for the development of disease. The incomplete concordance observed in monozygotic twin studies across various autoimmune conditions, including multiple sclerosis and rheumatoid arthritis, underscores the substantial role of non-genetic factors in initiating and shaping the clinical course of autoimmune diseases, including PV (14–17). Thus, the “exposome” takes center stage, encompassing a broad array of environmental exposures and life experiences, such as medications, infections, psychosocial stressors, dietary factors, immunizations, and physical insults, collectively shaping the complex etiology of PV (4, 5). Multiple reviews have confirmed that among the environmental factors associated with the onset of PV, drugs/pharmaceuticals are by far the most commonly reported and well characterized (18, 19). A recent review by our group found that drugs/pharmaceuticals accounted for 35% of all the literature regarding PV and environmental factors (20).

The first report of pemphigus triggered by pharmaceutical use stretches back over four decades to Degos in 1969 (21). Since then, an ever-growing amalgamation of agents reported to trigger and/or exacerbate pemphigus has accumulated in the literature. Those agents are often grouped based on shared biochemical or structural features, namely: the thiol group (captopril, gold sodium thiomalate, penicillamine, penicillin, etc.), the phenol group (rifampin, aspirin, etc.), and the non-thiol, non-phenol group (hydroxychloroquine, imiquimod, hydrochlorothiazide, irbesartan, nifedipine, nivolumab, etc.), discussed at length elsewhere (5, 18, 19). Among the diverse list of drugs associated with triggering PV only a single agent has exhibited a dramatic recent spike – hydroxychloroquine (non-thiol, non-phenol group (20)). The link between hydroxychloroquine and pemphigus is based on a single case report from 2006 (35), which, while valuable, is inherently limited in terms of the evidentiary weight it can offer by the anecdotal and idiosyncratic nature of case reports.

Hydroxychloroquine and chloroquine are members of the 4-aminoquinoline class of antimalarial agents (22). Today, hydroxychloroquine is included in the treatment guidelines for systemic lupus erythematosus (SLE), rheumatoid arthritis (RA), primary Sjögren syndrome, and antiphospholipid syndrome (23) and has been reported to be of benefit to a wide variety of diseases, detailed elsewhere (23–25).

In 2020, hydroxychloroquine found itself at the center of debate over its use in the prevention and/or treatment of the then novel COVID-19 pandemic (26–28).

Given the sudden prominence of hydroxychloroquine use in the setting of the COVID-19 pandemic, a previously documented case report of pemphigus triggered by hydroxychloroquine use, and the well-recognized role of drugs in the induction of autoimmune diseases we conducted a pharmacovigilance study to broadly assess the potential link between hydroxychloroquine use and the development of pemphigus using publicly available, FDA-generated data. We found statistically significant, disproportionally elevated reporting of the adverse event-drug combination of hydroxychloroquine-pemphigus, which constitutes a pharmacovigilance “signal” worthy of further investigation.

## 2 Methods

### 2.1 Study design and data sources

This observational, retrospective, pharmacovigilance analysis used the FAERS database to analyze the relationship between the adverse event of “pemphigus” and exposure to the drug hydroxychloroquine. We employed the validated pharmacovigilance tool OpenVigil 2.1 to query the FAERS database and perform disproportionality analysis. This study involves FAERS data from Q4 of 2003 to Q2 of 2023. Adverse events in FAERS, as queried in the present study, are reported in accordance with the Medical Dictionary for Regulatory Activities (MedDRA) version 24, as previously described (29).

### 2.2 Case selection and controls

Cases were identified by the presence of the MedDRA PT for “pemphigus” (10034280) and then sorted based on exposure to the drug of interest, hydroxychloroquine, or lack thereof. In pharmacovigilance, the traditional comparator or control used to determine whether the drug-event combination of interest is disproportionately overrepresented is “all-other-reports” of adverse events in the database, excluding the adverse event of interest (30–32).

We also conducted disproportionality analysis based on the combination of hydroxychloroquine and adverse events defined by the MedDRA PT “pemphigoid” (10034277), encompassing the related, though distinct, pemphigoid, sub-epidermal group of autoimmune blistering disorders which includes bullous pemphigoid.

### 2.3 Statistical analysis

Disproportionality analysis is based on the comparison of the proportion of adverse events for the drug of interest, here hydroxychloroquine, with the proportion of adverse events for all other drugs. Specifically, we calculated the Reporting Odds Ratio (ROR) defined in greater detail in **Supplementary Materials** under the heading “**Supplementary Methods**” and in **eTable 1**.

Determination of the statistical significance of a given signal can be made using three pieces of information: the lower bound of the 95% CI of the ROR (lbROR), the chi-squared value, and the absolute number of reports (n) with lbROR >1, and χ^2^ > 4, and n > 3 (30, 32).

Hydroxychloroquine exposure rates across the overall sample and by sex were analyzed using Jeffreys prior method to obtain 95% confidence intervals, and differences by sex were assessed with Fisher’s exact test (see **Table 1**). Pemphigus incidence related to hydroxychloroquine use was also evaluated across the sample and by sex cohorts, employing logistic regression to model the interaction between drug exposure and sex. The analysis aimed to assess hydroxychloroquine’s impact on pemphigus occurrence and to compare the drug-event relationship across sexes, deriving odds ratios and their 95% confidence intervals from the model (see **Table 2**). Analyses were performed using SAS v9.4.

**Table 1:** Odds of exposure to hydroxychloroquine by sex in FAERS.

Cohort	Rate of Hydroxychloroquine Exposure (95% CI)	Odds of Exposure to Hydroxychloroquine
Overall	0.56% (0.55 – 0.56)	0.00563
Female	0.77% (0.76 – 0.77)	0.00776
Male	0.27% (0.27 – 0.28)	0.00271
Gender Not Reported	0.45% (0.44 – 0.46)	0.00442

**Table 2:** Odds of developing pemphigus based on exposure to hydroxychloroquine and sex in FAERS.

Cohort	Rate of Pemphigus		OR (95% CI)	P-value
	With Hydroxychloroquine	Without Hydroxychloroquine		
Overall	2.43% (2.31-2.55)	0.01% (0.01-0.01)	282.6 (261.0-306.1)	< 0.001
Female	2.63% (2.49-2.78)	0.01% (0.01-0.01)	378.7 (339.0-423.1)	< 0.001
Male	0.04% (0.01-0.09)	0.01% (0.01-0.01)	3.6 (1.4-9.8)	< 0.001
Gender Not Reported	4.92% (4.43-5.46)	0.01% (0.01-0.01)	424.4 (353.8-509.1)	< 0.001

## 3 Results

### 3.1 A dramatic increase in absolute number of hydroxychloroquine-pemphigus reports occurred from 2020-2023

**Figure 1:**
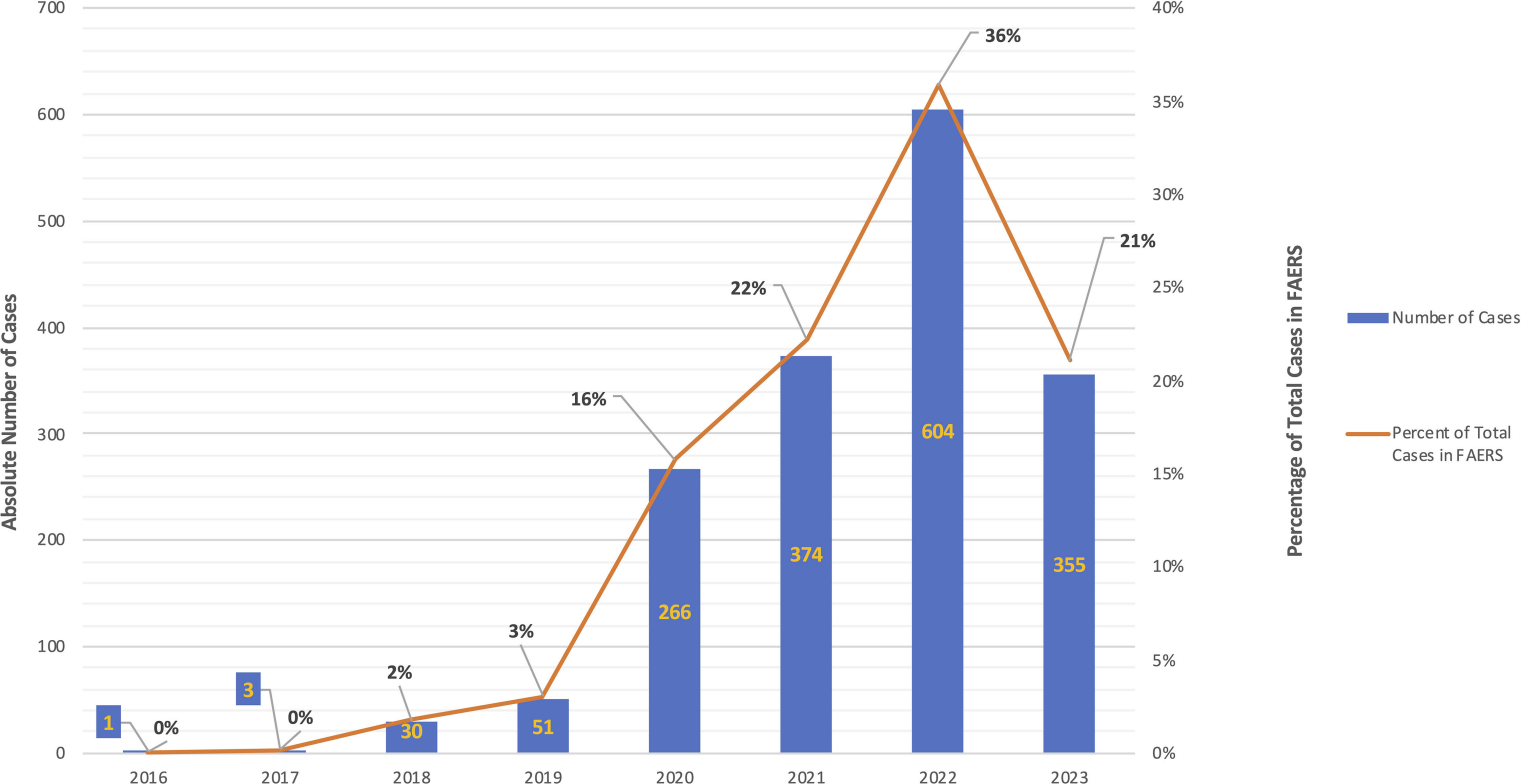
*Reports in FAERS with the combination of exposure to hydroxychloroquine and the adverse event pemphigus, by year.* Instances of the combination of exposure to hydroxychloroquine and the adverse event pemphigus reported to FAERS by year. Absolute number of cases are represented by blue bar graph (left axis) and the percentage of total cases per year are represented by the orange line graph (right axis). There were no reports of the combination hydroxychloroquine-pemphigus in the years 2003-2015 (not depicted).

We identified a total of 2,548 reports that included the adverse event pemphigus; among these, 1,545 reports (n=706 (41.92%) age 18-64, n=1 age 65-85 years, and n=977 (58.02%) with no age specified; n=1,366 (81.12%) females, n=4 (0.24%) males, and n=314 (18.65%) with no gender specified) included exposure to hydroxychloroquine (ROR, 282.647; 95% CI, 260.951-306.148). The highest number of cases was reported in 2022 (n = 604, 35.87%) (**Figure 1**), however, it must be noted that only data up to Q2 of 2023 were available for analysis at the time of authorship.

### 3.2 Hydroxychloroquine use and pemphigus represent a significant pharmacovigilance signal

In order to explore a potential association between hydroxychloroquine exposure and pemphigus we created a 2x2 contingency table of those two categorical variables (**Table 3**) (3, 30) and calculated a chi squared statistic with Yates’ correction as a precautionary measure to test for independence (χ^2^_Yates_ = 166,448.48) resulting in a hydroxychloroquine-pemphigus ROR and 95% confidence interval of 282.647 (260.951;306.148). This data suggests that the combination of hydroxychloroquine and the adverse event “pemphigus” likely represents a bona fide adverse drug reaction (as opposed to some combination of chance and background noise).

**Table 3:** Contingency table for hydroxychloroquine – pemphigus.

	Drug Exposure	No Drug Exposure	Sums
**Adverse Event Occurred**	1,545	1,003	2,548
**Adverse Event Did NOT Occur**	61,993	11,375,215	11,437,208
Sums	63,538	11,376,218	11,439,756

Accessed on 10/12/23 via OpenVigil 2.1.

### 3.3 Female sex is associated with significantly increased frequency of both hydroxychloroquine exposure overall, and subsequent development of the adverse event of pemphigus after exposure to hydroxychloroquine

The rate of hydroxychloroquine use is significantly different between females and males (p<0.001), with an odds ratio of 2.82 (95% CI: 2.76-2.88); individual odds ratios for exposure to hydroxychloroquine by sex in FAERS are provided in **Table 1**.

When comparing the risk of pemphigus between those with and without hydroxychloroquine exposure, significant differences are observed within all cohorts (all p<0.001). When comparing the effect of hydroxychloroquine on the risk of pemphigus between females (ROR 378.7 [339.0-423.01]) and males (ROR 3.6 [1.4-9.8]), there is a significant difference in the odds ratios (p<0.001). **Table 2** models the risk of developing pemphigus as a function of exposure to hydroxychloroquine, sex, and their two-way interaction.

### 3.4 Hydroxychloroquine use and pemphigoid do not represent a significant pharmacovigilance signal

In the interest of confirming that the adverse events included in the reports analyzed here were in fact those corresponding to *pemphigus* specifically and not to another autoimmune bullous disease, we also queried hydroxychloroquine use in bullous pemphigoid. χ^2^_Yates_ was 14.965. ROR and 95% confidence interval was **0.056** (0.008; 0.398). The total number of cases was 1. Overall, the combination of hydroxychloroquine and the adverse event of pemphigoid was *not* statistically significant according to the criteria given above.

## 4 Discussion

To the best of our knowledge, this is the first study to evaluate a relationship between hydroxychloroquine use and pemphigus via an analysis of real-world data from a large, population-based database. We described a significantly increased ROR for hydroxychloroquine and pemphigus well above both that observed for i) “all-other-reports” in FAERS as well as for ii) hydroxychloroquine and pemphigoid, our two comparators.

The pathogenesis of PV is a multifaceted process influenced by both genetic and environmental factors. However, the manifestation of clinically significant PV necessitates the intricate interplay of these genetic predispositions with additional non-genetic factors within the exposome (20). Among the diverse elements within the exposome, pharmaceuticals emerge as the most extensively studied environmental triggers in PV (19, 20). However, it must be noted that the overwhelming majority of the accounts linking particular environmental factors, including drugs, to PV are based on single case reports only (33–37). This does not discount the value of these reports, especially in a disease as rare as PV, but rather underscores the need for further investigation and potential confirmation of those reports using larger sample sizes and population level data. In 2006, Ghaffarpour et al. reported the case of a 52-year-old female who developed generalized blistering two weeks after the initiation of hydroxychloroquine to treat RA. The patient had not used any other drugs recently. Biopsy revealed suprabasal splitting and IgG/C3 depositions between keratinocytes. Notably, the patient reported a similar, albeit milder reaction about one month prior to a trial of chloroquine that resolved after initial discontinuation of the offending drug but recurred with “greater severity” after initiation of hydroxychloroquine. Following the results of the biopsy which were consistent with a diagnosis of PV, hydroxychloroquine was discontinued and the lesions resolved within about 3 weeks.

In support of this previous case report, our study reveals a significant increase in reports of the adverse event pemphigus among individuals exposed to hydroxychloroquine at the population level. Interestingly, we did not see the same association when analyzing the reports of adverse events of another autoimmune bullous disease, namely bullous pemphigoid. A wide range of drugs, including the gliptins, D-penicillamine, and nivolumab, among others, have been reported to be able to trigger bullous pemphigoid (38) however, to date, there are no reports in the literature detailing bullous pemphigoid induced by hydroxychloroquine. Notably, reports of other cutaneous pathologies associated with hydroxychloroquine including erythema multiforme (39), SJS (40)/TEN (41), and inverse psoriasis (42) can be found.

The present work has the advantage of being able to replicate the association reported in the 2006 case report in the context of a spontaneous reporting database with significantly larger sample sizes. While the methods of pharmacovigilance and disproportionality analysis cannot prove causality, they can provide an additional data point for clinicians and researchers alike to consider in their care and study of individuals with PV. The accumulation of such data points, and with them the development of a more sophisticated understanding of the pathomechanisms at play in PV, advances the goal of achieving personalized approaches to the management of each patient to better address the biological complexity underpinning the clinical heterogeneity of this disease.

Our findings demonstrate that spontaneous reports of the adverse event of pemphigus associated with the use of hydroxychloroquine significantly increased in the wake of the COVID-19 pandemic. This is paralleled by previous reports detailing a surge in prescriptions dispensed for hydroxychloroquine during the period from 02/16-04/25 2020 compared to 02/17-04/27 2019, with a total of 483,425 excess prescriptions filled during that period thought to be the result of off label prescriptions for COVID-19 (43). Moreover, we must consider that hydroxychloroquine was used off-label in combinations of drugs, at doses, and in populations that differ from its typical, studied uses and this too may have contributed to the observed increase in an otherwise uncommon adverse event such as pemphigus (44).

**Figure 2:**
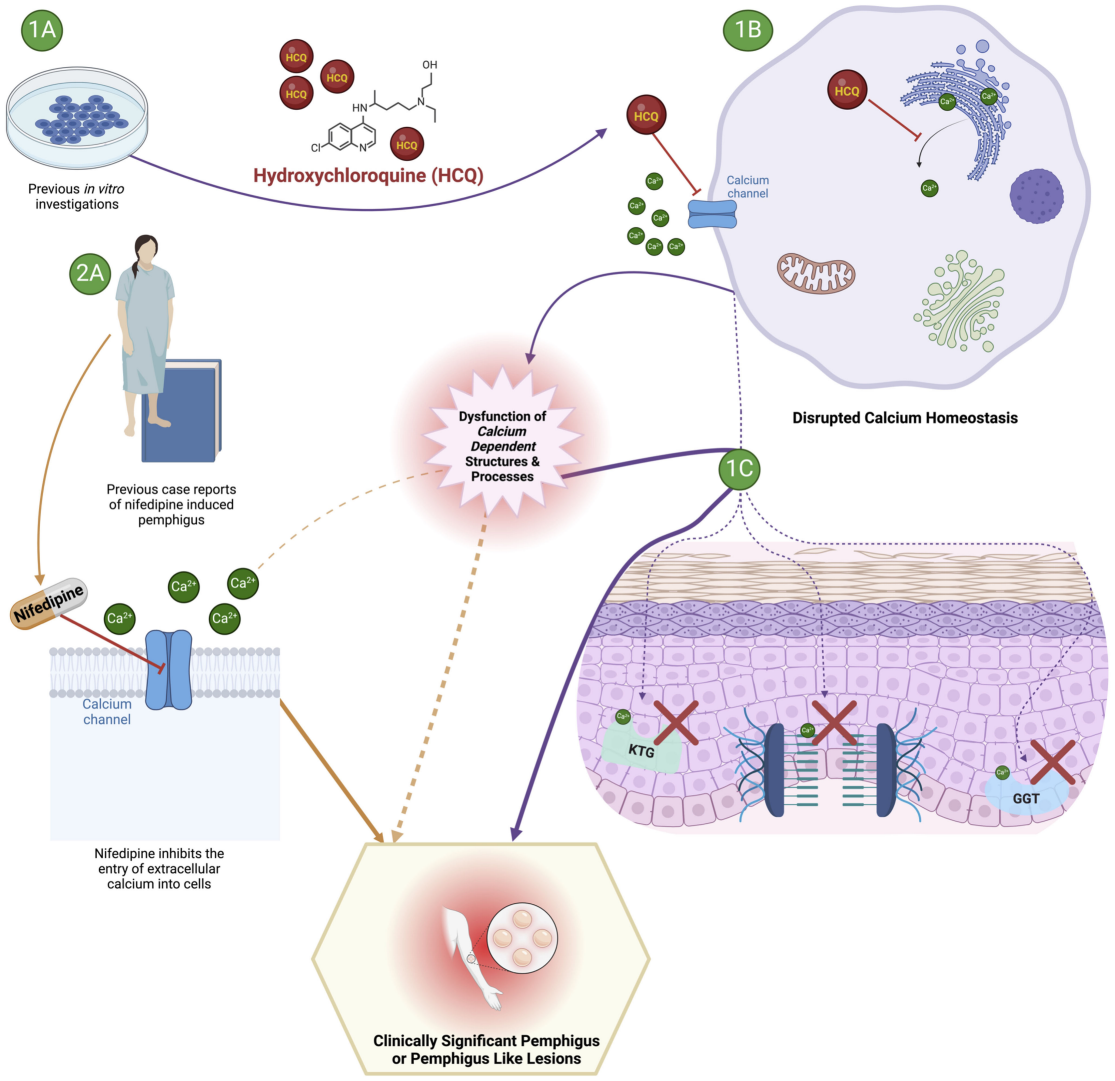
Proposed mechanism underlying the development of pemphigus or a pemphigus like syndrome following exposure to hydroxychloroquine. The effect of hydroxychloroquine may impact intracellular calcium homeostasis and subsequent disruption of calcium dependent structures and processes required to maintain epidermal integrity. 1A and 1B illustrate previously reported in vitro effects of hydroxychloroquine on calcium homeostasis leading to subsequent epidermal disruption illustrated in 1C (KTG, keratinocyte trans-glutaminase; GGT, gamma-glutamyl transpeptidase). 2A provides a clinical perspective to support the plausibility of a mechanistic link between exposure to a drug with effects on intracellular calcium homeostasis (nifedipine) and the development of pemphigus.

The exact pathophysiology underlying the increased risk of pemphigus development following exposure to hydroxychloroquine reported here have not been established. One plausible biological explanation linking hydroxychloroquine exposure to PV development centers on hydroxychloroquine’s effects on intracellular calcium homeostasis (22) (**Figure 2**). Hydroxychloroquine is thought to inhibit increases in intracellular calcium by both impeding the entry of extracellular calcium into cells, as well as by preventing the liberation of intracellular calcium stores (22). The conventionally recognized antigenic targets in the pemphigus group of diseases are desmoglein 3 (Dsg3) and desmoglein 1 (Dsg1), members of the calcium dependent cadherin superfamily of transmembrane cell adhesion molecules (11). Both Dsg3 and Dsg1 are essential components of the larger cell-cell adhesion structures known as desmosomes which join neighboring cells to one another and impart mechanical strength (45). As hydroxychloroquine is known to be able to alter the intracellular calcium milieu, it is conceivable that a downstream effect of those alterations would be a disruption of the aforementioned calcium dependent Dsg3 and Dsg1 structure/function, which could ultimately manifest in pemphigus or a pemphigus-like presentation. In earlier studies on drug induced pemphigus, Ruocco et al. demonstrated that keratinocytes deprived of calcium display decreased activity of enzymes required for keratogenesis (keratinocyte trans-glutaminase and gamma-glutamyl transpeptidase- both calcium dependent transpeptidases) as well as impaired desmosome formation, and ultimately cell-cell dyshesion, or acantholysis (46). The altered calcium explanation for the link between hydroxychloroquine and pemphigus is further bolstered by the fact that nifedipine, a calcium channel blocker that inhibits the entry of extracellular calcium into cells, has been shown *in vivo* and reported in multiple clinical cases to be able to both trigger and exacerbate pemphigus (47–50). One salient example is a case of pemphigus foliaceus reported by Kim et al. where disease relapse occurred in rapid succession following a clinical rechallenge with nifedipine, notably in a patient without any detected autoantibodies (50).

However, given the numerous reported potential mechanisms of action of hydroxychloroquine (22–24) additional pathways for the drug’s ability to induce PV among a subset of exposed individuals must also be considered. These may include hydroxychloroquine’s alkalization of endosomes and lysosomes (22–24) and the downstream effects of such pH changes on various immunologic signaling pathways including those mediated by toll like receptors (TLRs) (22–24) and chemokines such as CXCR4 (22) as well as on essential cellular processes such as autophagy, whose inhibition by hydroxychloroquine produces further still downstream effects on antigen presentation (22–24) which may ultimately interact with PV inductive mechanisms. Another potential mechanism of relevance may be related to hydroxychloroquine-mediated permeabilization of lysosomal, mitochondrial, and plasma membranes which facilitate signaling cascades resulting in cellular death (22, 51, 52). Finally, another plausible mechanism for the ability of hydroxychloroquine to induce PV may involve drug-mediated changes in the expression of cytokines such as IL-1, IL-6, TNF-α, and IFN- γ in mononuclear cells (53) and TNF-α, IFN-α, IL-6, and CCL-4 by plasmacytoid dendritic cells (54, 55) together with the subsequent alterations of immunologic signaling pathways regulated by those cytokines.

Despite the wide range of proposed mechanisms, it is widely accepted that the net effect of hydroxychloroquine treatment is immuno*modulation* rather than more conventional immuno*suppression*, as with drugs such as methotrexate or mycophenolate mofetil. This is supported by the fact that hydroxychloroquine use is *not* associated with increased risk of either infectious complications or cancer, as is the case with traditional immunosuppression (23). While this is certainly of benefit to those patients prescribed hydroxychloroquine for the treatment of RA or SLE, it is conceivable that very same modulation of the immune system may, in other individuals, tip the immunological equilibrium toward the development of a Th2 mediated disease like pemphigus.

Despite the lack of definitive evidence for any one particular mechanism of action, the strong statistical evidence for the association between hydroxychloroquine use and the development of pemphigus reported here corroborates the previous case report-based evidence that hydroxychloroquine likely represents a trigger factor for the development of pemphigus.

This retrospective, pharmacovigilance study has several limitations. Individual reports in FAERS are not validated by the FDA before their inclusion in the database (2, 56). Moreover, individual reports may vary substantially in their quality, with some lacking detailed information about drug dosing, patient demographics, or the adverse event itself (2, 56). These limitations were perhaps most elegantly explained by Sakaeda et al. who wrote that “a report in the FAERS database is a story, sometimes only a rumor, but numerous reports can reflect reality” (3). The adverse events reported in FAERS cannot be definitively attributed to a given drug exposure (56). Overall, adverse events are believed to be widely underreported in spontaneous reporting systems like FAERS, estimated at only about 6% and variable based on the particular adverse event (3, 57). On the other hand, it is possible that reporting of adverse events may be increased after a particular drug or adverse event is highlighted among the general public. This phenomenon, known as notoriety bias, is a form of reporting bias (57). While it is conceivable that the reporting of adverse events related to hydroxychloroquine may have been impacted by the widespread media coverage around it specifically and the COVID-19 pandemic more broadly, there had been no previous association between hydroxychloroquine and *pemphigus* specifically, save for the single aforementioned 2006 case report. Moreover, both Neha and Hoffman have previously shown that notoriety bias does not exist in FAERS and measures of disproportionality are not affected by safety alerts from the FDA (58, 59).

As has been previously stated, pharmacovigilance analyses such as this are suitable for hypothesis generation only and cannot, on their own, prove causality. COVID-19 has itself been documented to have an impact on both cutaneous disease generally, and on pemphigus more specifically with numerous case reports detailing pemphigus triggered or exacerbated by either COVID-19 infection or vaccination against the same (33, 60–66). COVID-19 infection and vaccination therefore both represent potential confounding factors in the present work.

## Data Availability

Publicly available datasets were analyzed in this study. This data can be found here: https://openvigil.sourceforge.net/#.
