# Supplementary material for: Evaluation of link between COVID-19 adjacent spike in hydroxychloroquine use and increased reports of pemphigus: a disproportionality analysis of the FDA Adverse Event Reporting System
Source: Front Immunol. 2024 Dec 20;15:1470660. doi: 10.3389/fimmu.2024.1470660 (PMC11695399; doi:10.3389/fimmu.2024.1470660)
Supplement: Supplementary file 1 [file Table1.docx]

**Supplementary Materials:**

**Supplementary Methods:**

**“Cleaning” of FAERS Data**

OpenVigil 2.1 was designed for the purpose of performing pharmacovigilance complete case analysis and is superior to using data directly from FAERS itself as OpenVigil “cleans” FAERS data by removing duplicate and incomplete reports^1^. The data in FAERS is said to be “dirty” in that it includes not only the aforementioned duplicate and incomplete reports but also reports with non-understandable drug names ^1^. These non-understandable drug names are a result of the fact that the FAERS data for drug names is a verbatim free text string that allows for entry of text that may include spelling errors, trade names, and/or abbreviations. To overcome this, OpenVigil 2.1 utilizes a mapping logic based on the external drug databases drugbank.ca and drugs@FDA, and only loads fully understandable reports (i.e complete case analysis). Under this paradigm, approximately 67.5% of the total number of reports on FAERS are loaded into OpenVigil 2.1^1^.

While the cleaned data from OpenVigil 2.1 (n=1545) were used to conduct statistical/disproportionality analysis, the uncleaned data (i.e. the data obtained directly from FAERS) were used to produce the summary characteristics for the set of all reports of pemphigus as an adverse event associated with hydroxychloroquine use during the period encompassing Q4 2003 until Q2 2023.

While the statistical, or disproportionality, analysis performed in this work are based only on the “cleaned” data from OpenVigil 2.1 with n=1545 “cases” (i.e the combination of the drug of interest (hydroxychloroquine) and adverse event of interest (pemphigus), the summary/characteristics of the cases reported in the results section are based on the *uncleaned* data directly from FAERS as OpenVigil 2.1 does not make such summary data available. Because the summary data are based on direct FAERS data, the total number of cases, n=1684, is greater than the number of cases available in OpenVigil/used in disproportionality analysis by 139 (1684-1545 = 139)^2^ .

**Statistical Analysis**

The ROR is the ratio of the odds of the adverse event of interest occurring with the drug of interest compared to the odds of the same event occurring with all other drugs in the database^3,4^. The null or expected value for the ROR is 1 and the values calculated for a given combination of drug and event provide information about the strength of the association between the two, with ROR behaving similarly to an odds ratio (the higher the value, the greater the strength of the signal/association) ^5^. Thus, if the proportion of the adverse event of interest (pemphigus) is greater in those exposed to hydroxychloroquine versus not exposed to hydroxychloroquine, an association can be hypothesized between the drug and the event and is considered a disproportionality signal^5^. Generally, the higher the value of a measure of disproportionality, the more likely a true association between the given drug and adverse event has been found. However, this association can only be verified through future research that is able to establish causality, which the present approach *cannot*.

Measures of statistical association or independence are calculated using a chi-square test with one degree of freedom and Yates’ correction. χ^2^_Yates_ is given by: N * ( | DE*de – dE*De | - N/2 )2 / (D * d * E * e).

It should be noted that the formulas used by OpenVigil, also reported here, were successfully validated against R/epiR 0.9-69 and web based contingency table calculators, as reported by Böhm et al in 2016.

**eTable 1: 2x2 Contingency Table Layout**

|  | **Drug Exposure** | **No Drug Exposure** | **Sums** |
| --- | --- | --- | --- |
| **Adverse Event Occurred** | DE | dE | E |
| **No Adverse Event Occurred** | De | de | e |
| **Sums** | D | d | N |

*Capital letters indicate the occurrence of either drug exposure (D) or adverse event (E). Lowercase letters indicate no drug exposure (d) or adverse event (e) occurred.*

The Odds Ratio is adapted in pharmacovigilance studies as the Reporting Odds Ratio (ROR) given by:

ROR = ( DE / De ) / ( dE / de ) = DE*de / De*dE

In **Table 2**, the rate of hydroxychloroquine exposure in FAERS is summarized in the overall sample and by sex using 95% confidence intervals obtained by Jeffreys prior method. The rates are compared between levels of sex using Fisher’s exact test, with odds ratios presented with 95% confidence intervals. The rate of hydroxychloroquine exposure is significantly different between females and males (p<0.001), with an odds ratio of 2.82 (95% CI: 2.76-2.88). The rate of hydroxychloroquine exposure is significantly different between females and gender not reported (p<0.001), with an odds ratio of 1.71 (95% CI: 1.67-1.75). And lastly, the rate of hydroxychloroquine exposure is significantly different between males and gender not reported (p<0.001), with an odds ratio of 0.61 (95% CI: 0.59-0.62).

In **Table 3**, the rate of reporting the adverse event pemphigus is summarized by exposure to hydroxychloroquine in the overall sample and within each sex cohort using 95% confidence intervals obtained by Jeffreys prior method. The pemphigus status is modeled as a function of hydroxychloroquine exposure, sex, and their two-way interaction term using a logistic regression model. Tests about the appropriate contrasts of model estimates are used to: a) evaluate the impact of hydroxychloroquine exposure on the occurrence of pemphigus within each cohort, and b) compare this relationship between sexes. From the model estimates odds ratios and corresponding 95% confidence intervals are obtained.

**Supplemental References:**

1. Böhm R, Bulin C, Waetzig V, Cascorbi I, Klein HJ, Herdegen T. Pharmacovigilance-based drug repurposing: The search for inverse signals via OpenVigil identifies putative drugs against viral respiratory infections. *Br J Clin Pharmacol*. 2021;87(11):4421-4431. doi:10.1111/bcp.14868

2. Center for Drug Evaluation and Research. FDA Adverse Event Reporting System (FAERS) Public Dashboard. FDA. Published October 22, 2021. Accessed October 12, 2023. https://www.fda.gov/drugs/questions-and-answers-fdas-adverse-event-reporting-system-faers/fda-adverse-event-reporting-system-faers-public-dashboard

3. Böhm R. Primer on Disproportionality Analysis. Published online October 16, 2018.

4. Böhm R, von Hehn L, Herdegen T, et al. OpenVigil FDA - Inspection of U.S. American Adverse Drug Events Pharmacovigilance Data and Novel Clinical Applications. *PLoS One*. 2016;11(6):e0157753. doi:10.1371/journal.pone.0157753

5. Sakaeda T, Tamon A, Kadoyama K, Okuno Y. Data Mining of the Public Version of the FDA Adverse Event Reporting System. *Int J Med Sci*. 2013;10(7):796-803. doi:10.7150/ijms.6048
